# Has the ‘Fast-Track’ referral system affected the route of presentation and/or clinical outcomes in patients with colorectal cancer?

**DOI:** 10.1186/s12957-016-0911-8

**Published:** 2016-06-08

**Authors:** Luke Thornton, Harriet Reader, Stevan Stojkovic, Victoria Allgar, Nick Woodcock

**Affiliations:** Hull York Medical School, York University Heslington, York, YO10 5DD UK; Department of General Surgery, York Hospital, York Teaching Hospital NHS Foundation Trust, Wigginton Road, York, YO31 8HE UK

**Keywords:** Colorectal, Cancer, General, Surgery, Outcomes, Survival

## Abstract

**Background:**

The aim of this study is to determine whether the ‘Fast-Track’ referral system has changed the route by which patients present with colorectal cancer (CRC) and whether the route of presentation has any effect on clinical outcome.

**Methods:**

A retrospective cohort study of patients diagnosed with CRC under the care of two consultant colorectal surgeons between April 2006 and December 2012. The route by which patients presented was categorised as Fast-Track (FT), non-Fast-Track (non-FT) or acute. Outcome variables were operative intent, disease stage and 2- and 5-year survival.

**Results:**

A total of 558 patients were identified. One hundred ninety-seven patients (35.3 %) were referred as FT, 108 (19.4 %) presented acutely and 253 patients (45.3 %) presented via other routes (non-FT)*.* Over the study period, the route of presentation did not change significantly (*P* = 0.135). There was no significant difference between FT and non-FT groups in terms of the proportion of patients undergoing potentially curative surgery (70.6 vs 74.3 %, *P* = 0.092) or with node-negative disease (48.2 vs 52.2 %, *P* = 0.796) nor was there any difference in 2-year or 5-year survival (74.1 vs 73.9 %, *P* = 0.837 and 52.3 vs 53.8 %, *P* = 0.889, respectively). Patients who presented acutely were less likely to undergo curative resection, had more advanced disease and had worse 2- and 5-year survival.

**Conclusions:**

The Fast-Track referral system has not affected the route by which patients present with CRC nor has it had any effect on clinical outcomes. Alternative strategies are required if the desired improvement in outcomes is to be achieved.

## Background

Colorectal cancer (CRC) is the fourth most common cancer and the second commonest cause of cancer-related death in the UK, with over 40,000 new cases and approximately 16,000 deaths per year [1–3]. The current 5-year survival in the UK is 54.4 % in men and 55.1 % in women, but as high as 90 % if diagnosed at its earliest stage (Dukes’ A) [4]. This compares unfavourably with elsewhere in Europe, where survival rates are 8–12 % higher [5]. This disparity is perceived as being due to a reluctance of patients to consult their GP with bowel-related symptoms, delays in referral of patients from primary to secondary care and/or delays in both investigation and subsequent treatment once referred [6].

**Fig. 1:**
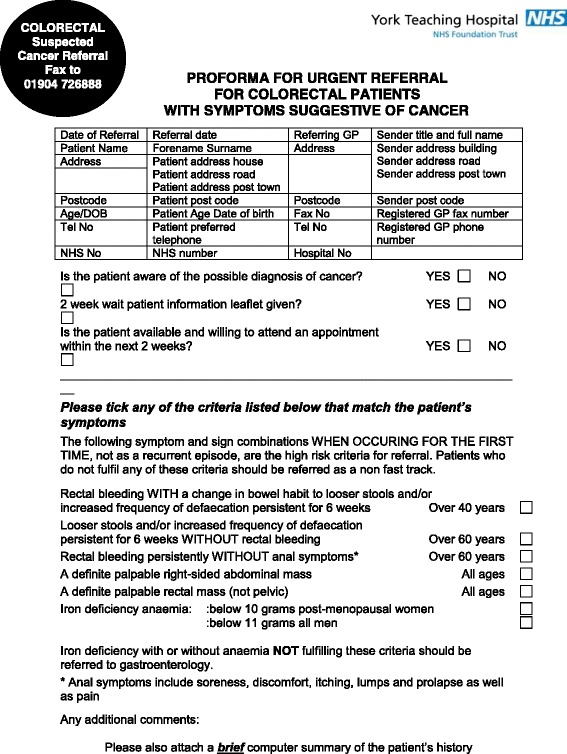
Fast-Track referral proforma

In 2000, the UK Department of Health introduced the ‘Fast-Track’ or ‘Two Week Wait’ system for patients with suspected cancer [7], in an attempt to address some of these issues. This system applies to all the common cancers, including CRC. For patients fulfilling any of the agreed referral criteria, the GP completes a standardised referral proforma (Fig*.*1). Patients referred in this way and then have to be seen in secondary care within 14 days of referral, and if they are subsequently diagnosed with CRC, they have to commence treatment within 62 days of the original GP referral. These targets have inevitably exerted a considerable strain on resources in secondary care. Further, a large proportion of patients with CRC present via other routes: non-Fast-Track referrals to the colorectal clinic and other specialties, e.g. Gastroenterology, the National Bowel Cancer Screening Programme and emergency admissions.

The aims of this study were to (1) determine whether the Fast-Track system has changed the route by which patients present with CRC and (2) assess whether the route of presentation has any effect on outcomes, in terms of treatment intent, disease stage or survival.

## Methods

All patients diagnosed with CRC under the care of two consultant colorectal surgeons between April 2006 and December 2012 were identified from the responsible consultants’ personal prospective databases. For each patient, the following variables were recorded: age, gender, route of referral, treatment intent (curative or palliative), disease stage, and survival at 2 and 5 years. All research carried out is in compliance with the Helsinki Declaration. Study is approved by the York District Hospital Clinical Effectiveness Team.

### Statistical analysis

Chi-squared tests were used to compare categorical variables and ANOVA or independent *t* tests for continuous variables. Kaplan-Meier curves were undertaken for survival data, with a log-rank test to compare referral groups. Cox regression was used to investigate survival, adjusting for age, sex and stage. All analyses were undertaken on SPSS (v20). A *P* value of <0.05 was considered to indicate statistical significance.

## Results

A total of 558 patients with newly diagnosed CRC were identified, of which 310 patients (55.6 %) were male, with a median age of 73 years (IQR 64–80 years). Overall, 197 patients (35.3 %) were referred as a Fast-Track (‘FT’ group), 108 (19.4 %) presented acutely (‘acute’ group) and 253 patients (45.3 %) presented via other routes (‘non-FT’ group) (Table 1)*.* The relatively small number of patients diagnosed through the National Bowel Cancer Screening Programme is explained by the fact that it commenced in York only in February 2010.

**Table 1:** Route of presentation

Route of presentation	No. of patients (%)
Fast-Track	197 (35.3 %)
Non-Fast-Track	253 (45.3 %)
Gastroenterology	112
Colorectal clinic	87
NBCSP	21
Other	33
Acute	108 (19.4 %)

**Fig. 2:**
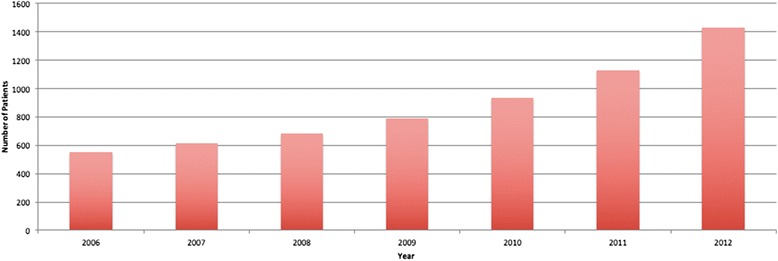
Number of Fast-Track referrals per year

**Fig. 3:**
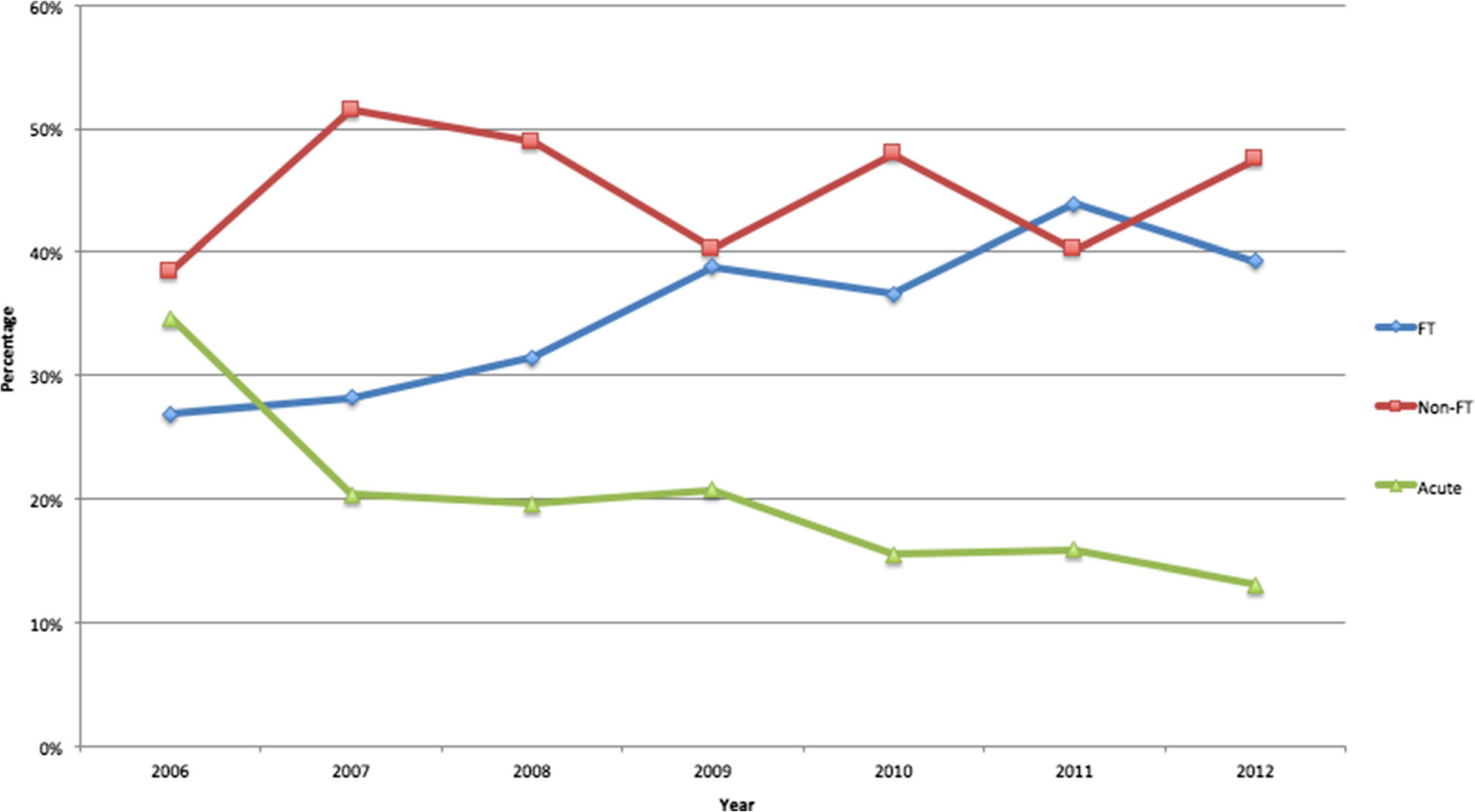
Changes in the route of presentation over time

The numbers of Fast-Track referrals per year are shown in Fig*.*2. There was an almost threefold increase from 551 in 2006 to 1425 in 2012. Over this time, there did appear to be a slight increase in the proportion of CRC patients referred as Fast-Tracks, with a concomitant reduction in numbers of patients presenting acutely, though these changes were not statistically significant (*P* = 0.135) (see Fig*.*3).

A similar proportion of patients in the FT and non-FT groups underwent potentially curative surgery (70.6 vs 74.3 %, *P* = 0.092), whereas significantly fewer acute patients (50.9 %) underwent curative surgery (*P* < 0.001). Similarly, equivalent proportions of FT and non-FT patients had node-negative disease (48.2 vs 52.2 %, *P* = 0.796), whereas the proportion of acute patients with node-negative disease (27.8 %) was significantly lower (*P* < 0.0001).

**Fig. 4:**
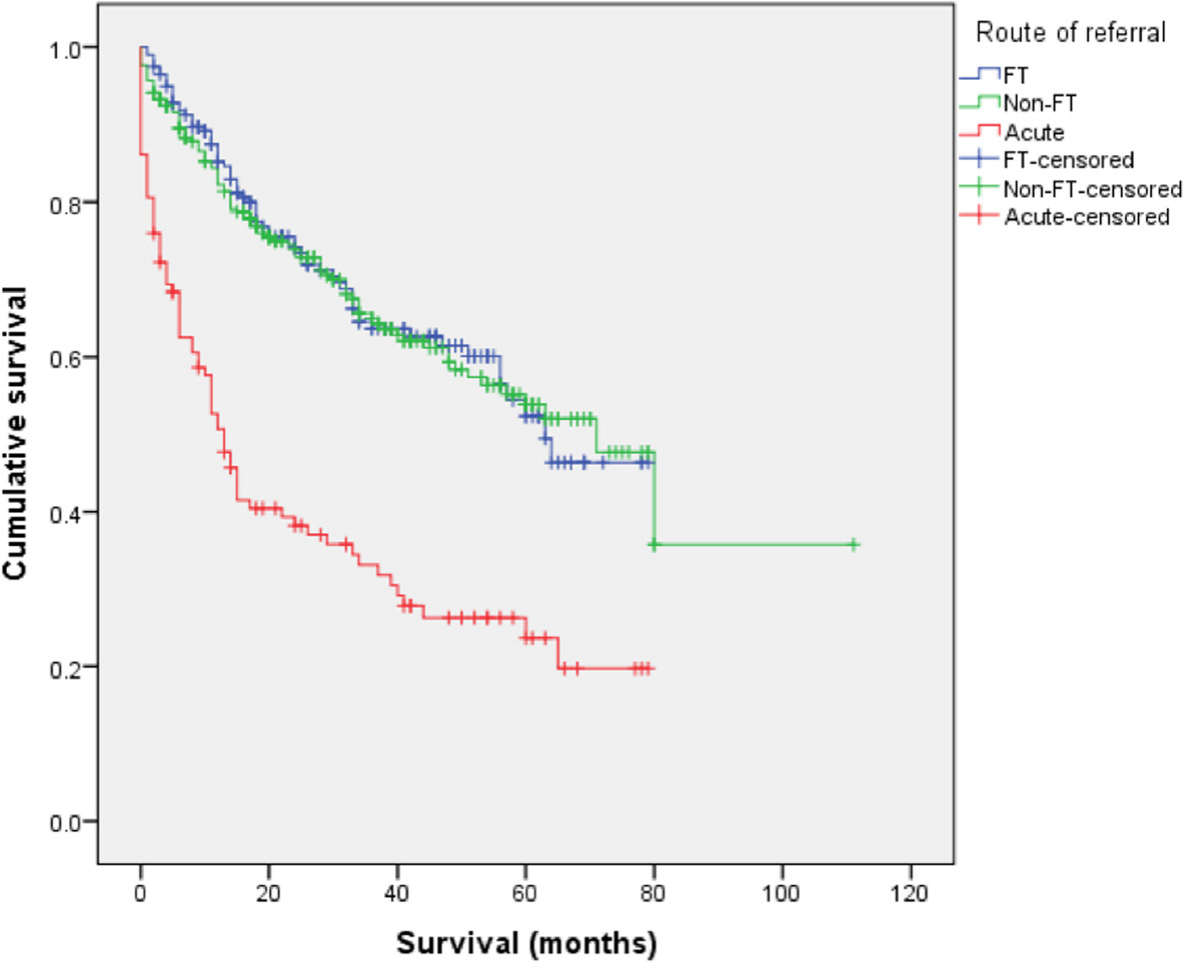
Cumulative survival

The overall mean survival for all patients was 57.8 months. Mean survival in the FT group was 52.7 months, compared to 64.1 months in the non-FT group (*P* = 0.912). However, mean survival in the acute patients was significantly lower (28.0 months, *P* < 0.001). Cumulative survival for each of the groups is shown in Fig*.*4. There was no significant difference between the FT and non-FT groups in terms of either 2-year overall survival (74.1 vs 73.9 %, *P* = 0.837) or 5-year overall survival (52.3 vs 53.8 %, *P* = 0.889). However, both 2- and 5-year overall survival were significantly lower in the acute group (38.2 %, *P* < 0.001 and 23.7 %, *P* < 0.001, respectively).

## Discussion

This study has shown that the Fast-Track system has not affected the route by which patients present with colorectal cancer. In particular, there has been no reduction in the proportion of patients presenting acutely. Further, CRC patients referred as a Fast-Track appear to do no better in terms of curative surgery, disease stage or survival when compared to non-Fast-Track patients, whereas those patients undergoing emergency surgery do significantly worse.

The Fast-Track system was introduced to guarantee that patients with symptoms suggestive of CRC are seen and investigated promptly when referred by their general practitioner. Once diagnosed, the patients are subsequently treated sooner than they would otherwise have been, with the expectation that this will improve outcomes. There has been an inexorable rise in the number of Fast-Track referrals over the last few years: in the current study, there was an almost threefold increase between 2006 and 2012. This has placed an inevitable strain on resources in secondary care. Having to process such large numbers of referrals in a timely manner has encouraged hospital trusts to adopt various investigative models, such as ‘straight-to-test’. Such pathways are largely dictated by the needs of the local population and the resources available. In York, all Fast-Track referrals are seen in a designated clinic within 2 weeks, where they are assessed by a surgeon, and a decision made regarding the most appropriate investigation(s).

It is important to remember that only about 10 % of Fast-Track patients actually have CRC [8, 9], and in the current study, only just over a third of CRC patients were referred as a Fast-Track. With such emphasis placed on meeting the Fast-Track waiting time targets, it is vital that this is not to the detriment of the majority of CRC patients who are not referred as a Fast-Track, whose investigations and/or treatment could otherwise potentially be delayed [10].

Bevis et al. similarly reported no significant differences between Fast-Track and non-Fast-Track patients in terms of the proportion of patients undergoing potentially curative surgery or disease stage [11], whereas other studies found that Fast-Track patients actually had more advanced disease [8, 9]. Further studies looking at survival found no difference at either 2 years [12] or 5 years [13]. Zafar et al. compared overall 5-year survival prior to and following the introduction of the Fast-Track system and found no difference [14].

There are various possible reasons as to why the Fast-Track system appears to be failing to achieve its aim of improving outcomes. The first possibility is that patients are being referred inappropriately. To increase sensitivity, the referral criteria are necessarily rather vague and open to interpretation. This inevitably reduces specificity, with consequent referral of significant numbers of patients with functional bowel disease and the ‘worried well’. Further, many patients do not actually fulfil the referral criteria. Chohan et al. found that only 73 % of Fast-Track referrals fulfilled the criteria, whereas 92 % of the patients subsequently diagnosed with CRC did so [8]. Conversely, patients with CRC who do fulfil the Fast-Track criteria frequently are not referred accordingly [15].

Patients referred via the Fast-Track route are undoubtedly investigated more quickly and undergo treatment sooner, but the difference is relatively small. In the study by Zafar et al., the median time from referral to treatment prior to the introduction of the Fast-Track system was 115 days, compared to 76 days after [14]. Another study comparing Fast-Track and non-Fast-Track patients demonstrated a significant reduction in both time to be seen by a colorectal specialist (median 31 vs 69 days) and time to initiation of treatment (median 42.5 vs 57.5 days) [11]. Whilst these differences are statistically significant, such modest reductions in time to treatment are unlikely to have a clinically significant impact on outcome.

Arguably more important delays occur prior to referral to secondary care. Patients are often reluctant to consult their GP with bowel-related symptoms, due to embarrassment, fear or ignorance. A recent study reported that 61 % of patients with rectal bleeding had thoughts about cancer, compared with only 29 % of those without rectal bleeding [16]. Alternatively, there may be a failure on the part of the GP to refer. Khattak et al. found that 79 % of patients who presented acutely with CRC had previously consulted their GP with symptoms, and in 38 % of all patients, it was more than 6 weeks before they were referred [17]. A further study looking at delays in diagnosis for various cancer types found breast cancer to have the shortest delay in presentation and suggested that this may be due to more obvious symptoms that are easily understood by patients, together with a high-profile national screening programme [18]. If this is true, then the National Bowel Cancer Screening Programme of faecal occult blood testing, plus impending flexible sigmoidoscopy screening, should hopefully serve to increase public awareness of the disease. Further, the recent ‘Be Clear on Cancer’ campaign, consisting of adverts on television, radio and in national newspapers and magazines, was introduced to raise public awareness of the symptoms of the disease, encourage patients to consult their GP and increase referrals to secondary care.

The most effective way of improving clinical outcomes must be to reduce the number of patients presenting as an emergency with CRC, in whom both short- and long-term outcomes are significantly worse. In the current study, we observed no significant change in the proportion of patients presenting acutely, despite a massive increase in the numbers of patients being referred as Fast-Tracks. This concurs with the latest National Bowel Cancer Audit Annual Report, in which the proportion of patients presenting acutely with CRC was 20.6 %, a figure that has remained frustratingly static over the last few years [19].

It would have been useful to be able to compare outcomes to those achieved prior to the introduction of the Fast-Track system in 2000, but the two surgeons whose patients are included did not commence their posts until 2006, so this data was not available. However, it is not unreasonable to presume that pre-2000 outcomes would have been comparable to the non-FT patients. We recognise that the patients included in the study constitute only a proportion of the total number of CRC patients managed by the unit, but as all newly diagnosed patients are allocated equitably between each of the surgeons, the patients studied should represent a true cross section of the total, with no referral bias. A further limitation of the current study is that we do not know the total number of non-FT referrals, but the numerous and varied routes by which patients can present with CRC means that this is almost impossible to ascertain. We do not have any data as to the predictive power of various symptoms in predicting the likelihood of a patient having CRC, but this was not the aim of the study. Some patients referred as a Fast-Track will inevitably be found to have other significant pathology, both malignant and benign, e.g. inflammatory bowel disease, but the primary reason for a Fast-Track referral must be a strong suspicion of CRC.

## Conclusions

In conclusion, this study has shown that the Fast-Track system has not affected the route by which patients present with CRC nor has it had any impact on outcomes. This is likely to be due to a combination of inappropriate referrals, delays in referral and failure of patients to seek medical attention with symptoms. Furthermore, it is unlikely that shortening a patient’s investigative and treatment pathway by a few weeks at most will make any difference to their outcome. Continued efforts should be directed at raising public awareness of the symptoms of bowel cancer and strongly encouraging patients to consult their GP with such symptoms.
